# Mechanical and Wetting Properties of Ta_2_O_5_ and ZnO Coatings on Alloy Substrate of Cardiovascular Stents Manufactured by Casting and DMLS

**DOI:** 10.3390/ma15165580

**Published:** 2022-08-14

**Authors:** Diana-Irinel Băilă, Răzvan Păcurar, Tom Savu, Cătălin Zaharia, Roxana Trușcă, Ovidiu Nemeș, Filip Górski, Ancuța Păcurar, Alin Pleșa, Emilia Sabău

**Affiliations:** 1 Department of Manufacturing Engineering, Faculty of Industrial Engineering and Robotics, University Politehnica of Bucharest, Blv. Splaiul Independenței, No. 313, Sector 6, 060042 Bucharest, Romania; 2 Department of Manufacturing Engineering, Faculty of Industrial Engineering, Robotics and Production Management, Technical University of Cluj-Napoca, Blv. Muncii, No. 103-105, 400641 Cluj-Napoca, Romania; 3 Advanced Polymer Materials Group, Department of Bioresources and Polymer Science, University Politehnica of Bucharest, 1-7 Gh. Polizu Street, 011061 Bucharest, Romania; 4 Department of Science and Engineering of Oxide Materials and Nanomaterials, Faculty of Applied Chemistry and Materials Science, University Politehnica of Bucharest, Blv. Splaiul Independenței, No. 313, Sector 6, 060042 Bucharest, Romania; 5 Department of Environmental Engineering and Sustainable Development Entrepreneurship, Faculty of Materials and Environmental Engineering, Technical University of Cluj-Napoca, Blv. Muncii, No. 103-105, 400641 Cluj-Napoca, Romania; 6 Faculty of Mechanical Engineering, Poznan University of Technology, 60-965 Poznan, Poland; 7 Department of Mechatronics and Machine Dynamics, Faculty of Automotive, Mechatronics and Mechanical Engineering, Technical University of Cluj-Napoca, Blv. Muncii, No. 103-105, 400641 Cluj-Napoca, Romania

**Keywords:** thin films, DMLS process, contact angles, mechanical simulations, cardiovascular stents

## Abstract

In the last years, additive manufacturing technologies have been developed, especially direct metal laser sintering, and used in the dental and medical implant domains. Cardiovascular stents have evolved from bioinert, bare metal cages to biomimetic devices that promote tissue regeneration or healing. In this paper, comparisons concerning mechanical properties between Co–Cr alloy and cast 304L stainless steel were realized using FEM analysis, necessary for manufacturing cardiovascular stents by DMLS technology using Co–Cr alloy. The purpose of this paper consists of the evaluation of the contact angle at the interface of the Co–Cr alloy manufactured by DMLS, respectively, cast stainless steel 304L, and thin film deposition realized by the e-gun method (Ta_2_O_5_ and ZnO). Scanning electronic microscopy SEM and EDX techniques were employed for morphological investigation of the sintered samples manufactured by the DMLS process. They were also used for semi-quantitative and qualitative chemical and metallographic analyses. The e-gun coating was used to obtain thin films with the nanometer order of Ta_2_O_5_ and ZnO with a protective role to improve the corrosion resistance, roughness, and antiseptic role.

## 1. Introduction

Additive manufacturing technology has realized a real medical revolution due to the manufacturing possibilities and the various materials that can be used, from plastic, ceramic to metallic alloys. These technologies permit the manufacture, in a very short time, of various special prostheses with complex shapes, personalized implants or customized devices for each patient depending on the disease. This technology is used frequently in the dental, orthopedic and in surgery domains [1,2,3].

Cardiovascular stents are used in angioplasty surgery and are used to open blocked coronary arteries caused by coronary artery disease. Angioplasty is recommended in an emergency setting such as a heart attack. Pan. C. considered that, in angioplasty, a catheter, a long and thin tube with a tiny balloon at its tip, is introduced into a blood vessel and localized to the blocked coronary artery. In the narrowed area of the heart artery, the balloon is inflated and presses the blood clot against the sides of the artery, allowing more space for blood flow, as remarked by Borhani, S. [4,5].

The cardiovascular stent is a tiny, expandable metal mesh coil that is put into the newly opened area to stop the narrowing or closing again. Once introduced, the cardiovascular stent will be coated by the tissue as a layer of skin. For this, the material used to manufacture the stents must be chemically inert and nontoxic for the human body [4,5,6,7].

There are two groups of stents, as stated earlier, self-expanding and balloon-expandable stents [4,5]. Early stents were mostly fabricated from metals (until very recently); hence the first-generation of stents are called bare-metal stents. These permanent metallic frameworks are made of stainless steel and cobalt–chromium (Co–Cr) alloys for balloon-expandable, and nickel–titanium alloys (nitinol) for self-expanding stents, as specified by Udriște A. [7].

In this paper, FEA analysis for cardiovascular stents, realized by cast 304L stainless steel and by Co–Cr alloy manufactured by DMLS, was conducted to establish the possibility of stent manufacturing by the new additive manufacturing technology, direct metal laser sintering (DMLS), after “stl” file for stent design. The FEA simulations were realized using Onshape software [8,9,10,11,12].

The direct metal laser sintering process uses a laser source with a high power to realize the binding metallic powder and construct the solid parts after a CAD model [13,14].

In this research, we conducted SEM and EDX analysis, mapping determination to establish the qualitative and quantitative chemicals contained, and the elemental distribution on the samples realized by casting technology for the cast 304L stainless steel samples and by the DMLS process for the superalloy Co–Cr samples [15,16,17,18,19,20]. 

Materials such as cast 304L stainless steel, 316L, Co–Cr alloys, and Ti alloys are frequently used in implant manufacturing because they are not toxic to the human body, have a good corrosion resistance, mechanical resistance, and allow for good cleaning (like glass) [21].

The aim of our work was to establish the contact angle at the interface of the Co–Cr alloy, respectively, of the cast 304L stainless steel samples and various oxides coatings with different concentrations (Ta_2_O_5_ and ZnO) to determine the comportment in the simulated biological fluid [22,23,24,25,26,27,28,29,30,31,32,33,34,35,36,37,38,39,40,41].

In this paper, samples of the cast 304L stainless steel and Co–Cr alloy were coated with thin films of Ta_2_O_5_ and ZnO in different concentrations by e-gun technology to improve the hardness, the roughness surface, the corrosion resistance, the bioactivity, and the biocompatibility. In the literature, the adhesion strength and the corrosion resistance of the coatings were examined by a micro-scratch tester and electrochemical workstations for the multilayer ZnO-Ta_x_O_y_ [37,38,39].

Therefore, the main objective of this article was focused on realizing FEA simulations to establish the mechanical characteristics of materials used for the manufacture of cardiovascular stents to conduct a comparison between the materials’ stent manufacture in the function of the technology used—casting and direct metal laser sintering technology—and to remark on the influence of the thin film coatings concerning the bioactivity and roughness aspects of the cardiovascular stents.

## 2. Materials and Methods

### 2.1. Co–Cr Samples Made by DMLS Process and Cast 304L Stainless Steel, Coated with Ta_2_O_5_/ZnO by E-Gun Technology

The materials used for the samples in this paper were Co–Cr powders (ST2724G), used for the manufacture of parts by the direct metal laser sintering (DMLS) process and the cast stainless steel 304L for medical use. The chemical composition of the Co–Cr alloy powder used in the DMLS process was: 54.31%Co; 23.08%Cr; 11.12% Mo, 7.85% W, 3.35% Si and Mn, Fe <0.1%. The mechanical properties of the Co–Cr alloy powder used for the DMLS process were: elastic limit 0.2% (Rp0.2) = 815 MPa; elongation at break = 10%; Vickers hardness = 375 HV 5; elastic module = 229 GPa; density = 8.336 g/cm^3^; corrosion resistance < 4 µg/cm^2^; thermal expansion coefficient = 14.5 × 10^−6^ K^−1^; Poisson’s ratio = 0.29–0.3 [35].

The samples realized by cast stainless steel 304L presented the following chemical composition: Fe balance; 17.5–20% Cr; 8–11% Ni; Mn <2%; Si <1%; C <0.8%; P <0.45%; S <0.3%. The mechanical properties of the cast stainless steel 304L were: density 7.85–8.06 g/cm^3^; modulus of elasticity = 190–203 GPa; tensile strength = 510–620 MPa; compressive strength = 205–310 MPa; elastic limit = 205–310 MPa; endurance limit = 175–260 MPa; electrical resistivity = 0.72 × 10^−6^ Ω·m, melting point = 1673–1723 °C, thermal conductivity = 16.2 W/m·K; thermal expansion = 17.2 × 10^−6^ K^−1^; Poisson’s ratio = 0.265–0.275 [12].

The cast stainless steel 304L was manufactured by hot rolled in plate with a thickness of 2 mm and a width of 500 mm by the Shangyi company (Wuxi City Jiangsu Province, China). For the experimental part, we used three cast stainless steel 304L samples and three samples of Co–Cr alloy manufactured by the DMLS process. The samples of Co–Cr had a thickness of 1 mm and a diameter of 10 mm and the samples of cast 304L stainless steel had a thickness of 2 mm and a diameter of 20 mm.

The sintered disks by the DMLS process were designed in the Solid Works 2018 program made by the Dessault Group ((Vélizy-Villacoublay, France) and saved as a “stl” file. For the DMLS process, we used a Phenix Systems dental machine, type PHX Pro200 Dental (Riom, France) with the following technical specifications: the fiber laser P = 50 W, λ = 1070 nm, manufactured volume is 100 × 100 × 80 mm, machine dimensions are L = 1.20 m; l = 0.77 m; H = 1.95 m [36]. 

The machine used Phenix Dental software, and the sintering process was realized at the temperature of 1300 °C and in a nitrogen atmosphere for 30 min. The scanning speed was 0.06 m/s, and the thickness of the deposited powder layer was 20 μm. The linear energy density (LED), which was defined by the ratio of laser power to the scanning speed, was used to tailor the laser sintering mechanism. The value of LED was between 3400 and 6000 J/m. After the sintering process, the samples were slowly air cooled. The samples were supported in a post treatment at 800 °C in the furnace for 30 min and slowly cooled in the air to obtain a better mechanical resistance. 

Then, the Co–Cr samples and 304L samples were mechanically polished and cleaned with distilled water and alcohol before coating deposition by e-gun technology. The samples were mounted on the dome chamber, cleaned in plasma glow discharges, and the coatings were realized with zinc oxide nanopowder (CAS No. 1314-13-2, Sigma-Aldrich (St. Louis, MO, USA), code 544906-10G) and tantalum (V) oxide 99% nanopowder (CAS No. 1314-61-0, Sigma-Aldrich (St. Louis, MO, USA), code 303518-25G). The nanopowders were compacted in the target form, necessary for e-gun technology. The deposition process was conducted in a physical vaporous deposition chamber in vacuum, with the pressure between 10^−5^ and 10^−6^ torr. The oxides were vaporized thermally by two electron guns, one for Ta_2_O_5_ and other for ZnO, in a high vacuum, which was necessary to reduce the contamination risk of the samples. To control the coating growth to report on the thickness and evaporating rate, we used a quartz crystal microbalance. 

To increase the density of the coating material, we used ion guns. Using an electron bombardment at 180 °C, the thermal evaporation determines the transition from a solid to vaporous state. The average speed for the coating films was between 10 and 100 A/s and the process occurred at 3000 °C.

### 2.2. Finite Element Analysis of the Cardiovascular Stents of Co–Cr Alloy Manufactured by DMLS Process and by Cast Stainless Steel 304L

The cardiovascular stents were designed using the Onshape program by Onshape (Cambridge, MA, USA). Onshape is a cloud CAD/CAM software and permits FEA simulations, rendering, and other cloud-based engineering tools.

### 2.3. Electronic Microscopy of Co–Cr Alloy and 304L Samples Coated with Ta_2_O_5_/ZnO by E-Gun Technology 

The SEM (scanning electron microscopy) and EDX (energy-dispersive X-ray spectroscopy) analysis of the samples were performed using a scanning electron microscope QUANTA INSPECT F type with a field emission gun (FE-SEM) (Hillsboro, OR, USA) and a resolution of 1.2 nm, coupled with an energy-dispersive X-ray spectrometer (Hillsboro, OR, USA) with a resolution of 133 eV at MnK. The areas of interest were analyzed qualitatively by microcompositional X-ray spectrometry.

### 2.4. Contact Angles Determination of Co–Cr Alloy and 304L Samples Coated with Ta_2_O_5_/ZnO by E-Gun Technology

The wettability was realized at the interface of the Co–Cr alloy and thin film deposition, respectively, at the interface of the cast 304L stainless steel and thin film deposition samples by using a contact angle apparatus made in Finland. 

To establish the surface and interfacial tension, the static and dynamic contact angles and the surface free energy of solids on the dried films were determined using KSV-CAM 101 apparatus. Ultrapure water droplets were used with a drop volume of 20 μL. 

For each contact angle, the measurement was made within 10 s after each drop to ensure that the droplet did not soak into the compact, and the contact angles reported were the mean of five determinations. The smaller contact angles corresponded to increased wettability. Distilled water was used to determine the hydrophilic character of the Co–Cr alloy used for the cardiovascular stents. Three specimens of the Co–Cr alloy manufactured by DMLS technology and coated with thin films with the nanometer order of Ta_2_O_5_/ZnO by e-gun technology were used. The other three samples of the cast 304L stainless steel coated with thin films of Ta_2_O_5_/ZnO were used for a comparison of the characteristics of the contact angles. The meniscus formed immediately after the surface was viewed through a stereoscopic microscope, and the optical beam was deflected horizontally by mirror precision. The resulting image was processed on computer software to measure the wetting angles.

## 3. Results and Discussions

### 3.1. Co–Cr Samples Made by DMLS Process and Cast 304L Stainless Steel, Coated with Ta_2_O_5_/ZnO by E-Gun Technology

**Figure 1:**
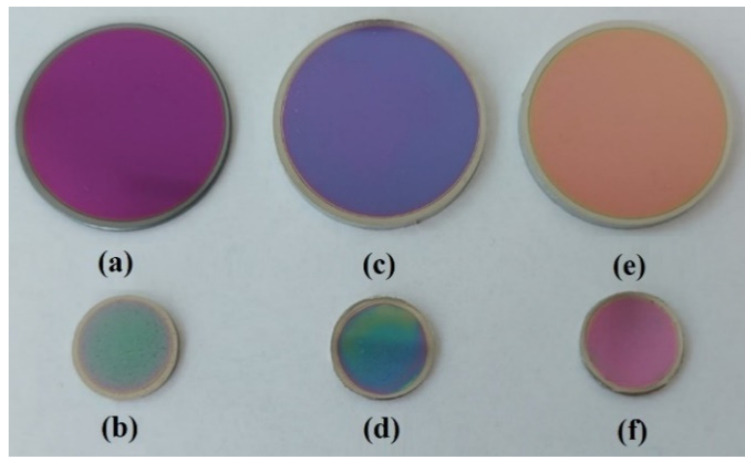
Samples coated with the thin films: (**a**,**b**) Samples S1 (cast 304L) and S2 (Co–Cr alloy sintered DMLS) coated with Ta_2_O_5_; (**c**,**d**) samples S3 (cast 304L) and S4 (Co–Cr alloy sintered DMLS) coated with 50% Ta_2_O_5_, 50% ZnO; (**e**,**f**) samples S5 (cast 304L) and S6 (Co–Cr alloy sintered DMLS) coated with 75% Ta_2_O_5_, 25% ZnO.

The six samples of the cast 304L stainless steel S1, S3, and S5 (shown in Figure 1a,c,e) and Co–Cr alloy samples S2, S4 and S6 (presented in the Figure 1b,d,f) sintered by the DMLS process were coated with thin films by e-gun technology, with a thickness of 15 microns. Samples S1 and S2 were coated with Ta_2_O_5_, and samples S3 and S4 presented a thin composite film of 50% Ta_2_O_5_ and 50% ZnO. Samples S5 and S6 had a coating composite film of 75% Ta_2_O_5_ and 25% ZnO.

### 3.2. Finite Element Analysis of the Cardiovascular Stents of Co–Cr Alloy Manufactured by DMLS Process and by Cast Stainless Steel 304L

**Figure 2:**
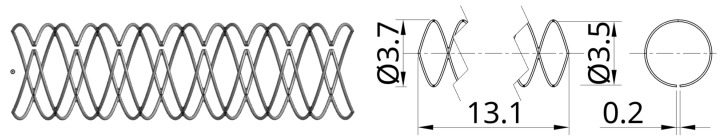
The cardiovascular stent 3D model and main dimensions (in mm).

For the analysis, a stent model was designed in Figure 2, where a 0.1 wire was used to build a double four revolutions helix with a 3.6 mm median diameter. The stent had a 0.2 mm cut, allowing for the stent radial stretching.

**Figure 3:**
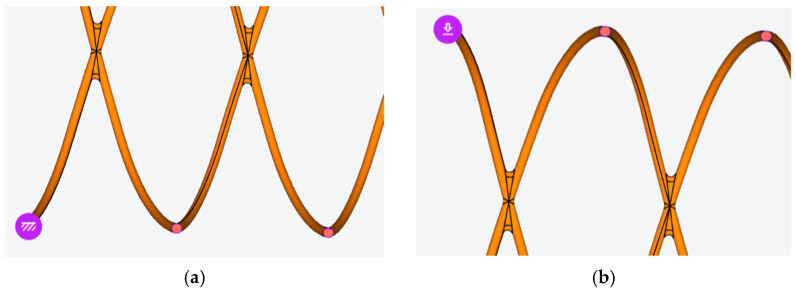
Restraint (**a**) and radial force constraints (**b**) applied on the stent model for the FEA simulation.

For the finite element analysis, the OnScale Solve cloud engineering simulation platform was used. Values of 8336 kg/m^3^, 0.295 and 229 GPa were used for the material’s mass density, Poisson’s ration, and Young’s modulus, respectively. For symmetry reasons, a longitudinal section was applied, and only half of the model was used in the simulation. A restraint type constraint was applied on half of the circular faces resulted from cutting the model, on the bottom side, and normal forces were also applied on the other circular faces, on the upper side, as shown in Figure 3.

Simulations were performed for different values of the normal forces and the results are shown in Table 1.

Third-order regression polynomials were determined for the displacement and von Mises stress:

(1) $$d\;\left[\mathrm{mm}\right]=-0.0005\cdot F^{3}+0.0058\cdot F^{2}+2.426\cdot F-0.0011$$

(2) $$\mathrm{stress}\;\left[\mathrm{GPa}\right]=0.0003\cdot F^{3}-0.004\cdot F^{2}+4.2324\cdot F-0.0011$$

**Figure 4:**
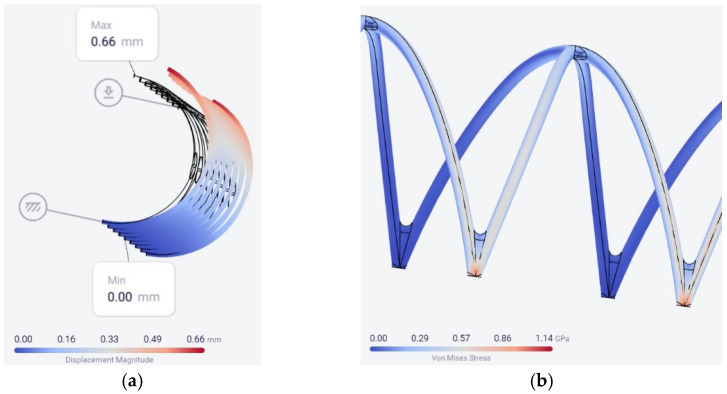
The location of the maximum values for the displacement (**a**) and von Mises stress for the Co–Cr alloy (**b**).

The simulation results for the 0.27 N force are shown in Figure 4.

Looking at the simulation data, the greatest von Mises stress, which was still less than the estimated tensile strength of 1.200 GPa of the Co–Cr alloy, meaning the value of 1.14 GPa obtained for the 0.27 N force, showed that the 0.66 mm (17.8% of the diameter) displacement was enough to ensure that the stent will remain fixed after stretching.

**Figure 5:**
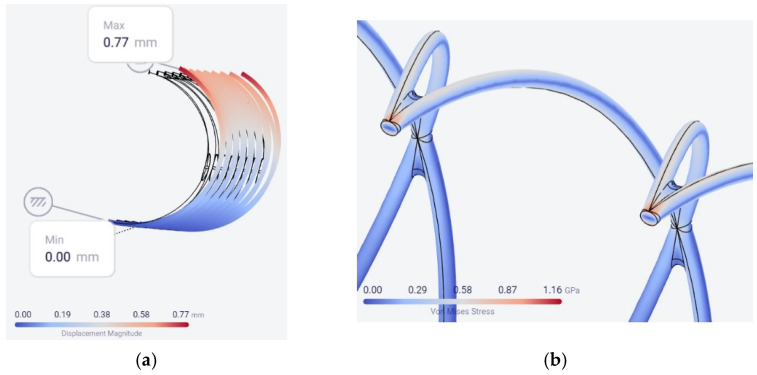
The location of the maximum values for the displacement (**a**) and von Mises stress for the cast 304L stainless steel (**b**).

The same model, with a force of 0.27 N and the same conditions, was used to simulate the behavior of a stent made from cast 304L stainless steel with a mass density of 7950 kg/m^3^, a Poisson’s ratio of 0.27, and a Young’s modulus of 196 GPa. As expected, the maximum displacement was a little higher (0.77 mm) than in the case of the Co–Cr alloy (0.66 mm) and the maximum von Mises stress was practically the same: 1.16 GPa for the 304L steel compared to 1.14 GPa for the Co–Cr alloy, as shown in Figure 5.

### 3.3. Electronic Microscopy of Co–Cr Alloy and 304L Samples Coated with Ta_2_O_5_/ZnO by E-Gun Technology 

**Figure 6:**
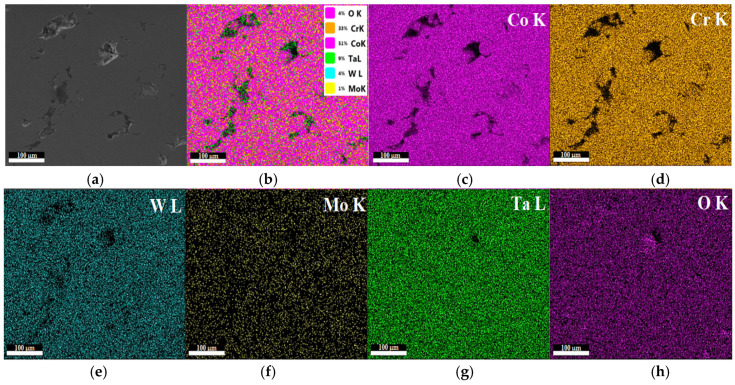
EDX mapping analysis of sample S2 of the Co–Cr alloy sintered by DMLS and coated with a Ta_2_O_5_ thin film; (**a**) SEM image and (**b**) the overall mapping elements on the same spot correspond to cobalt (**c**), chromium (**d**), wolfram (**e**), molybdenum (**f**), tantalum (**g**), and oxygen (**h**) mapping. Scale bar is 100 µm.

The morphological investigations and chemical elemental analysis of the six samples were realized by SEM, and for the three samples manufactured via DMLS, we conducted EDX mapping analysis. Concerning the mapping analysis, in Figure 6, we could observe a very thin uniform layer of Ta_2_O_5_ with fine equiaxed grains to the angstrom order presented on the sintered sample S2. The EDX mapping of the sintered sample S2 of the Co–Cr alloy and coated by e-gun technology with Ta_2_O_5_ is shown in Figure 6 and it can be seen that there was a major presence of CoK (51%), CrK (33%), WL (4%), MoK (1%), TaL (9%), and OK (4%). 

**Figure 7:**
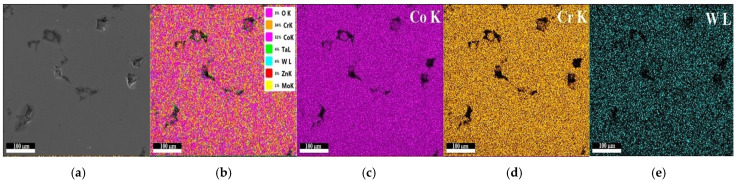

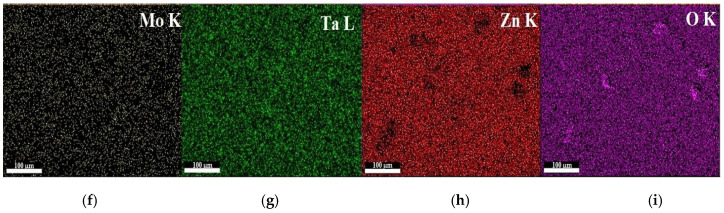
EDX mapping analysis for sample S4 of the Co–Cr alloy sintered by DMLS and coated with a thin film of 50% Ta_2_O_5_ and 50% ZnO; (**a**) SEM image and (**b**) the overall mapping elements on the same spot correspond to cobalt (**c**), chromium (**d**), wolfram (**e**), molybdenum (**f**), tantalum (**g**), zinc (**h**), and oxygen (**i**) mapping. Scale bar is 100 µm.

The EDX mapping analysis of the sintered sample S4 presented the uniform distribution of the thin composite film deposited (50% Ta_2_O_5_ and 50% ZnO) with fine grains in the nanometer order. The mapping analysis showed the chemical composition distribution of the sintered sample S4 with the following chemical elements: CoK (52%), CrK (34%), WL (4%), TaL (4%), ZnK (3%), MoK (1%), and OK (3%), as seen in Figure 7. 

**Figure 8:**
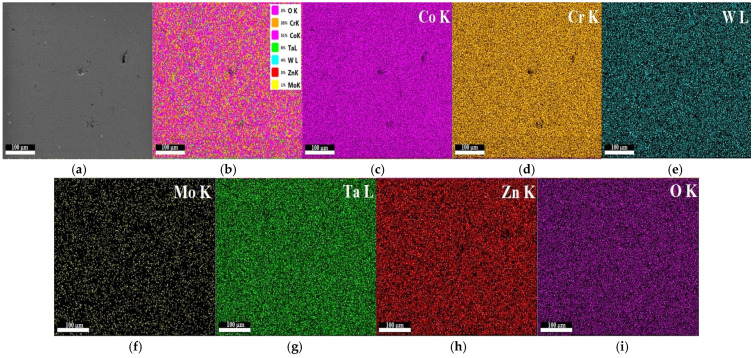
EDX mapping analysis for the sample S6 of Co–Cr alloy sintered by DMLS and coated with a thin film of 75%Ta_2_O_5_ and 25%ZnO; (**a**) SEM image and (**b**) Overall mapping elements on the same spot: corresponding to cobalt (**c**), chromium (**d**), wolfram (**e**), molybdenum (**f**), tantalum (**g**), zinc (**h**) and oxygen (**i**) mapping. Scale bar is 100 µm.

In Figure 8, the mapping of sintered sample S6 presents the uniform distribution of the thin composite film deposited (75% Ta_2_O_5_ and 25% ZnO) with fine grains in the nanometer order. The mapping analysis showed the chemical composition repartition of the sintered sample S6 with the following chemical elements: CoK (51%), CrK (33%), WL (4%), TaL (6%), ZnK (3%), MoK (1%), and OK (3%), as shown in Figure 8.

**Figure 9:**
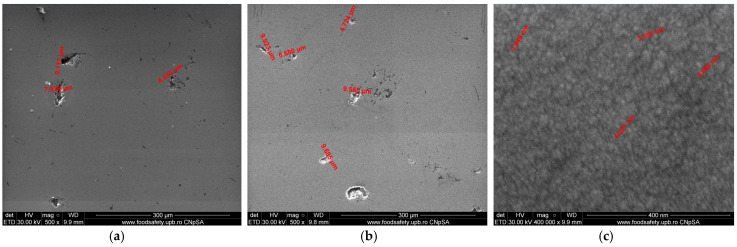
The FE-SEM analysis of the samples coated with a thin film of Ta_2_O_5_: (**a**) Sample S1 of the cast 304L stainless steel (500×). (**b**) Sample S2 of the Co–Cr alloy manufactured via DMLS (500×). (**c**) Thin film grains of Ta_2_O_5_ (400,000×).

Figure 9a shows the SEM analysis of the samples coated with a thin film of Ta_2_O_5_. For samples S1, it can be seen that there was a uniform thin layer deposition with some fine porosities of microns in the order between 4 and 9 µm, due to the substrate realized by the cast 304L stainless steel. The particle distribution of the chemical elements for the sample S1 was OK 23.44%, CrK 15.19%, FeK 51.71%, NiK 5.26%, and TaL 4.41%. Due to the substrate porosities, for sample S2, manufactured by the DMLS process using the Co–Cr alloy powder and coated with Ta_2_O_5_, it can be seen that there was a thin fine layer deposition and the appearance of several porosities of microns in the order between 6 and 10 µm (Figure 9b). The particle distribution of the chemical elements for sample S2 was OK 35.35%, CrK 17.2%, CoK 32.69%, TaL 3.68%, WL 1.77%, and MoK 9.3%. Figure 9c presents the thin grains of Ta_2_O_5_ with an equiaxial distribution of the angstrom order and average particle size between 3.5 and 4.5 nm. 

**Figure 10:**
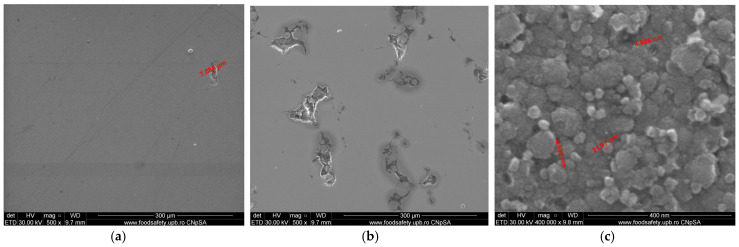
The FE-SEM analysis of the samples coated with a thin film of 50% Ta_2_O_5_ and 50% ZnO: (**a**) Sample S3 of the cast 304L stainless steel (500×). (**b**) Sample S4 of the Co–Cr alloy manufactured via DMLS (500×). (**c**) Thin film grains of 50% Ta_2_O_5_ and 50% ZnO (400,000×).

The SEM analysis of samples coated with a thin film of 50% Ta_2_O_5_ and 50% ZnO for sample S3 in Figure 10a showed a uniform thin layer deposition due of the substrate realized by the cast 304L stainless steel, with a small porosity of 7.08 µm. The particle distribution of the chemical elements for sample S3 was OK 20.43%, CrK 15.58%, FeK 54.21%, NiK 6.85%, TaL 1.87%, and ZnK 1.06%. Because of the substrate porosities for sample S4, manufactured by the DMLS process using Co–Cr alloy powder and coated with Ta_2_O_5_, it can be seen that there was a thin fine layer deposition and the appearance of greater porosities in the micron order, varying from 10 to 25 µm, as shown in Figure 10b. The particle distribution of the chemical elements for sample S4 was OK 28.99%, CrK 20.54%, CoK 41.85%, TaL 1.97%, WL 1.71%, ZnK 1.07%, and MoK 3.88%.

In Figure 10c, it can be observed that the thin composite grains of Ta_2_O_5_ and ZnO were in the nanometer order, and the average particle size was between 4.8 and 21.97 nm. 

**Figure 11:**
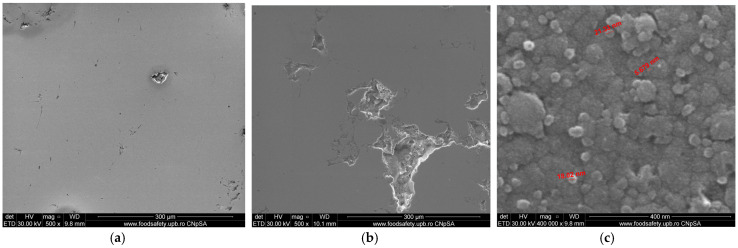
The FE-SEM analysis of samples coated with a thin film of 75% Ta_2_O_5_ and 25% ZnO: (**a**) Sample S5 of the cast 304L stainless steel (500×). (**b**) Sample S6 of Co–Cr alloy manufactured via DMLS (500×). (**c**) Thin film grains of 75% Ta_2_O_5_ and 25% ZnO (400,000×).

Concerning the SEM analysis of sample S5 coated with a thin film of 75% Ta_2_O_5_ and 25% ZnO, from Figure 11a, a uniform thin layer deposition could be observed due to the substrate realized by the cast 304L stainless steel, with small porosities between 4.54 and 9.11 µm. 

The particle distribution of the chemical elements for sample S5 was OK 19.33%, CrK 16.18%, FeK 55.58%, NiK 5.77%, TaL 2.18%, and ZnK 0.96%. 

Because of the substrate porosities for sample S6, manufactured by the DMLS process using Co–Cr alloy powder and coated with 75% Ta_2_O_5_ and 25% ZnO, it can be seen that there was a thin fine layer deposition and the appearance of greater porosities in the micron order, varying from 20 to 45 µm, as shown in Figure 11b. The particle distribution of the chemical elements for samples S6 was OK 34.05%, CrK 18.71%, CoK 37.47%, TaL 3.28%, WL 1.63%, ZnK 1.34%, and MoK 3.53%.

In Figure 11c, it can be seen that there were thin composite grains of Ta_2_O_5_ and ZnO in the nanometer order, the average particle size was between 5.67 and 21.33 nm, the Ta_2_O_5_ spherical grains were smaller and light grey, and the ZnO grains presented a greater spherical diameter and dark grey color.

**Figure 12:**
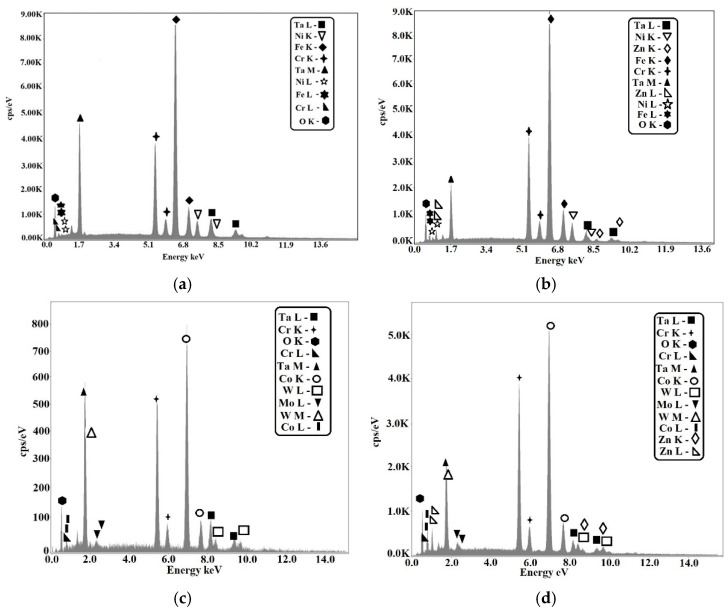
The EDX analysis of the samples: (**a**) S1 of cast 304L stainless steel coated with Ta_2_O_5_; (**b**) S3 of the cast 304L stainless steel coated with 50% Ta_2_O_5_ and 50% ZnO; (**c**) S2 of the Co–Cr alloy manufactured by DMLS and coated with Ta_2_O_5_; (**d**) S4 of the Co–Cr alloy manufactured by DMLS and coated with 50% Ta_2_O_5_ and 50% ZnO.

In Figure 12, the EDX analysis is presented for samples S1, S2, S3, and S4. For sample S1 (Figure 12a), the predominant peaks were Fe, Cr, Ni, and peaks also appeared for Ta and O, showing the presence of Ta_2_O_5_ in sample S1. In Figure 12b, for sample S3, predominant peaks in the 304L substrate such as Fe, Cr, Ni, but also Ta, Zn and O could be observed, indicating the presence of the composite film of both oxides 50% Ta_2_O_5_ and 50% ZnO. Concerning Figure 12c, the EDX analysis for sample S2 is shown, with major peaks of Co, Cr, W, Mo, respectively, and Ta and O peaks, showing the presence of Ta_2_O_5_ in the thin coating. In Figure 12d, for sample S4, peaks specific for the Co–Cr alloy substrate appeared and the thin film revealed the presence of the Ta, Zn, and O peaks, specific for the composite thin film coating of the two oxides. In this case, the XRD analysis showed that there were no clear peaks belonging to the thin layer. All visible peaks were also present in the substrate, which resulted in the layers being amorphous.

### 3.4. Contact Angles Determination of Co–Cr Alloy and 304L Samples Coated with Ta_2_O_5_/ZnO by E-Gun Technology

The contact angles were determined in the laboratory under proper conditions on the clean solid surfaces of the samples and using distilled water. The curvature of the drop is given by Young–Laplace equation and is nonlinear. For the 3D shape of the drop, we used the energy minimization method. The wettability analysis is very sensitive to chemical compounds of the sample and can show the variation in the values obtained.

(3) $$K_{m}=\frac{1}{2}*\frac{\left(1+f_{x}^{2}\right)*f_{yy}-2*f_{x}*f_{y}*f_{xy}+\left(1+f_{y}^{2}\right)*f_{xx}}{{\left(1+f_{x}^{2}+f_{y}^{2}\right)}^{\frac{3}{2}}}\;$$

**Figure 13:**
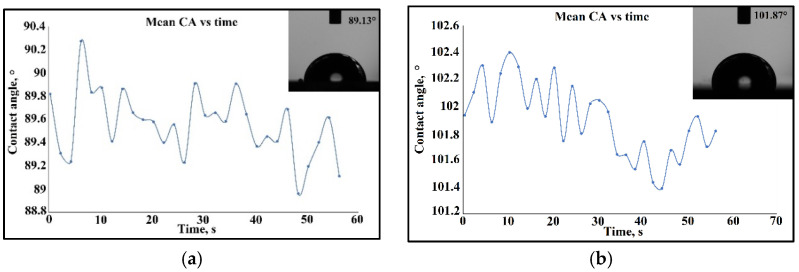
The contact angle measurement of the samples coated with a thin film of Ta_2_O_5_ by e-gun technology: (**a**) Sample S2 of the Co–Cr alloy fabricated via the DMLS process; (**b**) Sample S1 of the cast 304L stainless steel.

**Figure 14:**
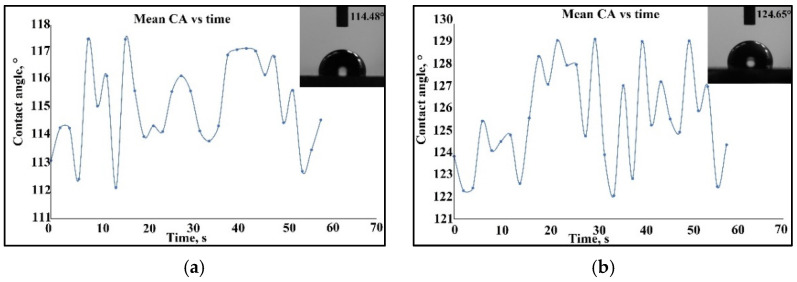
The contact angle measurement of the samples coated with a composite film of 50% Ta_2_O_5_ and 50%ZnO by e-gun technology: (**a**) Sample S4 of the Co–Cr alloy fabricated via the DMLS process; (**b**) Sample S3 of the cast 304L stainless steel.

In Figure 13a, for sample S2 of the Co–Cr alloy fabricated via DMLS and coated with Ta_2_O_5_ by e-gun technology, the drop shape presented an angle measurement close to 89.8°, and after stabilization of the curvature angle in time, the angle reached 89.13°. In the case of Figure 13b, for sample S1 of the cast 304L stainless steel coated with Ta_2_O_5_ by e-gun technology, the contact angle was nearly 101.9°, then after stabilization of the curvature angle in time, the angle reached 101.87°. In this case, it can be seen that the influence of the substrate concerned the bioactivity of the samples. The samples realized by Co–Cr alloy manufactured by DMLS and coated by Ta_2_O_5_ had a hydrophilic character in rapport with the sample fabricated by the cast 304L stainless steel, due to the porosity of the substrate and perhaps of capillary phenomena. For sample S4 of the Co–Cr alloy fabricated via DMLS and coated with 50% Ta_2_O_5_, 50% ZnO by e-gun technology, the contact angle was nearly 113°, and after a minute, it stabilized at 114.48°, as shown in Figure 14a. For sample S3 of the cast 304L stainless steel coated with 50% Ta_2_O_5_ and 50% ZnO by e-gun technology, the wettability was 124° and stabilization was realized at 124.65°, as seen in Figure 14b.

The influence of the substrate could also be observed, as seen in the samples realized by the Co–Cr alloy manufactured by DMLS and coated by Ta_2_O_5_, which presented a hydrophilic property in rapport to the sample fabricated by the cast 304L stainless steel. 

Furthermore, the influence of the contact angle determination in the function of the chemical composition of the thin composite film of 50% Ta_2_O_5_ and 50% ZnO, where the appearance of ZnO on the substrate increased the angle contact and the sensitivity of the contact angle determination, presenting a hydrophobic characteristic.

**Figure 15:**
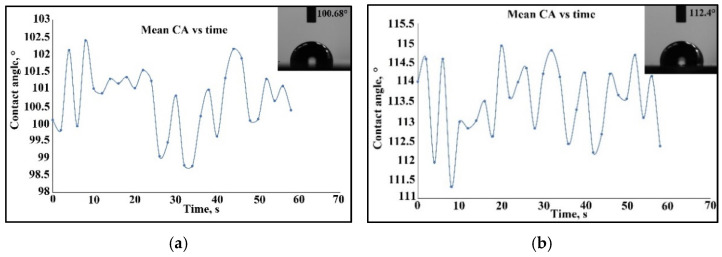
The contact angle measurement of the samples coated with a composite film of 75%Ta_2_O_5_ and 25%ZnO by e-gun technology: (**a**) Sample S6 of the Co–Cr alloy fabricated via the DMLS process; (**b**) Sample S5 of the cast 304L stainless steel.

For sample S6 of the Co–Cr alloy manufactured via the DMLS process and coated with a thin composite film of 75% Ta_2_O_5_ and 25% ZnO by e-gun technology, the contact angle began at 100.2° and the stabilization took place at 100.68°, as shown in Figure 15a. In Figure 15b, for sample S5, the wettability determination for the cast 304L stainless steel coated with a thin composite film of 75% Ta_2_O_5_ and 25% ZnO by e-gun technology started at 114° and the stabilization was realized after 60 s at 112.4°. The thin film of Ta_2_O_5_ presented a hydrophilic character rather than the composite film of Ta_2_O_5_ and ZnO. It was also observed that the Co–Cr sample had a pronounced hydrophilic property in support of the sample of the cast 304L stainless steel.

## 4. Conclusions

The idea of cardiovascular stents has revolutionized the treatment of coronary diseases, where stents were used in surgery for the first time for balloon angioplasty. Surgical stents have always presented a medical and technological challenge, both in terms of the materials that can be used as well as the design and manufacturing technologies.

The originality of this paper consists of the design and the FEA simulation to establish the mechanical properties of the Co–Cr alloy used in the DMLS process, which is necessary to manufacture personalized cardiovascular stents, the comparison with the cast 304L stainless steel and the sophisticated coating with thin films was realized to improve the roughness and the bioactivity. In this paper, we obtained comparisons between two materials that can be used for cardiovascular stent manufacture—cast 304L stainless steel and Co–Cr alloy used in DMLS manufacture—using SEM and EDX analysis, respectively, to measure the contact angles. 

Generally, stents are tubular implants that give stenotic arteries or other non-vascular conduits mechanical strength until the risk of full closure be removed and for this, in this paper, we designed a cardiovascular stent and realized different FEA mechanical simulations for the cardiovascular stent using Onshape software for both materials. The cast 304L stainless steel permitted a greater displacement (0.77 mm) than the Co–Cr alloy (0.66), presenting approximately the same maximum von Mises stress, showing that both materials can be used safely in stent fabrication. In this paper, we determined the EDX mapping analysis for the Co–Cr alloy samples sintered by DMLS and coated with various thin film of Ta_2_O_5_ and ZnO by e-gun technology to improve the roughness, the corrosion resistance, the hardness, the bioactivity, and the biocompatibility. Realizing the contact angle determination, which is a sensitive analysis, can provide insights into the characteristic pronounced hydrophilic of the Co–Cr alloy sintered by DMLS as a substrate, due to the porosities of the structure obtained through this manufacturing process, in rapport by the samples realized by the cast 304L stainless steel. The contact angle values of the six samples determined the chemical homogeneity and smooth topography. 

The microstructure of the samples coated by e-gun technology with a thin film of Ta_2_O_5_ was very fine, homogenous, and uniform and the grains were of the angstrom order; for the composite film of Ta_2_O_5_ and ZnO, the grains were equiaxial of the nanometer order. It can be seen that the substrate had a particularly large influence, especially for samples of the Co–Cr alloy because of the porosities in the structure obtained after DMLS manufacture, the thin film deposed by e-gun technology presented the small porosities in the micron order than on the samples of the cast 304L stainless steel substrate. The contact angle determination is very important in characterizing the cell–biomaterial interactions and in establishing 3D printing processes. 

In the future, a great challenge will be to fabricate personalized medical stents from the Co–Cr alloy manufactured by the DMLS process with a small device profile design, thin strut thickness, and that is self-expanding, in order to realize a biomimetic and biodegradable coating, respectively, and a drug-eluting polymer coating on cardiovascular stents.

## Figures and Tables

**Table 1:** The simulated displacements and von Mises stresses.

Force [N]	Displacement [mm]	Von Mises Stress [GPa]
0.04	0.09738	0.17
0.10	0.24	0.42
0.27	0.66	1.14
0.70	1.70	2.96
2.00	4.87	8.45
10.00	24.34	42.23

## References

[1] Revilla-León M., Meyer M.J., Özcan M. (2019). Metal additive manufacturing technologies: Literature review of current status and prosthodontic applications. Int. J. Comput. Dent..

[2] Kuncická L., Kocich R., Lowe T.C. (2017). Advances in metals and alloys for joint replacement. Prog. Mater. Sci..

[3] Ferraiuoli P., Taylor J.C., Martin E., Fenner J.W., Narracott A.J. (2017). The accuracy of 3D optical reconstruction and additive manufacturing processes in reproducing detailed subject-specific anatomy. J. Imaging.

[4] Borhani S., Hassanajili S., Tafti S.H.A., Rabbani S. (2018). Cardiovascular stents: Overview, evolution, and next generation. Prog. Biomater..

[5] Pan C., Han Y., Lu J. (2021). Structural Design of Vascular Stents: A Review. Micromachines.

[6] Hoare D., Bussooa A., Neale S., Mirzai N., Mercer J. (2019). The future of cardiovascular stents: Bioresorbable and integrated biosensor technology. Adv. Sci..

[7] Udriste A.S., Niculescu A.G., Grumezescu A.M., Bădilă E. (2021). Cardiovascular stents: A review of past, current, and emerging devices. Materials.

[8] Alghazzawi T.F. (2016). Advancements in CAD/CAM technology: Options for practical implementation. J. Prosthodont. Res..

[9] Strub J.R., Rekow E.D., Witkowski S. (2006). Computer-aided design and fabrication of dental restorations: Current systems and future possibilities. J. Am. Dent. Assoc..

[10] Wang Y., Chen X., Zhang C., Feng W., Zhang P., Chen Y., Huang J., Luo Y., Chen J. (2019). Studies on the performance of selective laser melting porous dental implant by finite element model simulation, fatigue testing and in vivo experiments. Proc. Inst. Mech. Eng. H.

[11] Cosma C., Teusan C., Gogola P., Simion M., Gabalcova Z., Trif A., Berce P., Balc N. (2022). Investigation of the Interface between Laser-Melted CoCr and a Stainless-Steel Substrate. Metals.

[12] Raab A.G., Raab G.I., Tokar A.A., Rybalchenko O., Belyakov A., Dobatkin S.V., La P.Q. (2020). Microstructure and Mechanical Properties of AISI 304L Austenitic Stainless Steel Processed by Various Schedules of Rolling. J. Phys. Conf. Ser..

[13] Koutsoukis T., Zinelis S., Eliades G., Al-Wazzan K., Al Rifaiy M., Al Jabbari Y.S. (2015). Selective laser melting technique of Co-Cr dental alloys: A review of structure and properties and comparative analysis with other available techniques. J. Prosthodont..

[14] Kim H.R., Jang S.H., Kim Y.K., Son J.S., Min B.K., Kim K.H., Kwon T.Y. (2016). Microstructures and mechanical properties of Co-Cr dental alloys fabricated by three cad/cam-based processing techniques. Materials.

[15] Videršcak D., Schauperl Z., Šolic S., Catic A., Godec M., Kocijan A., Paulin I., Donik C. (2021). Additively Manufactured Commercial Co-Cr Dental Alloys: Comparison of Microstructure and Mechanical Properties. Materials.

[16] Hong J.H., Yeoh F.Y. (2020). Mechanical properties and corrosion resistance of cobalt-chrome alloy fabricated using additive manufacturing. Mater. Today Proc..

[17] McGrory B.J., Ruterbories J.M., Pawar V.D., Thomas R.K., Salehi A.B. (2012). Comparison of surface characteristics of retrieved cobalt-chromium femoral heads with and without ion implantation. J. Arthroplast..

[18] Sedlak J., Ptackova M., Nejedly J., Madaj M., Dvoracek J., Zouhar J., Charvat O., Piska M., Rozkosny L. (2013). Material analysis of titanium alloy produced by Direct Metal Laser Sintering. Int. J. Met..

[19] Reclaru L., Ardelean L.C. (2018). Current Alternatives for Processing CoCr Dental Alloys Lucien.

[20] Hunt J.A., Callaghan J.T., Sutcliffe C.J., Morgan R.H., Halford B., Black R.A. (2005). The design and production of Co-Cr alloy implants with controlled surface topography by CAD-CAM method and their effects on osseointegration. Biomaterials.

[21] Fu W., Liu S., Jiao J., Xie Z., Huang X., Lu Y., Liu H., Hu S., Zuo E., Kou N. (2022). Wear Resistance and Biocompatibility of Co-Cr Dental Alloys Fabricated with CAST and SLM Techniques. Materials.

[22] Hardness of Coatings on Medical Implants. https://www.helmut-fischer.com/hardness-of-coatings-on-medical-implants.

[23] Antanasova M., Kocjan A., Kovač J., Žužek B., Jevnikar P. (2018). Influence of thermo-mechanical cycling on porcelain bonding to cobalt–chromium and titanium dental alloys fabricated by casting, milling, and selective laser melting. J. Prosthodont. Res..

[24] Rupp F., Gittens Rolando A., Lutz S., Marmur A., Boyan B.D., Schwartz Z., Geis-Gerstorfer J. (2014). A review on the wettability of dental implant surfaces I: Theoretical and experimental aspects. Acta Biomater..

[25] Hacini N., Ghamnia M., Dahamni M.A., Boukhachem A., Pireaux J.J., Houssiau L. (2021). Compositional, structural, morphological, and optical properties of ZnO thin films prepared by PECVD technique. Coatings.

[26] Chang Y.Y., Lai C.H., Hsu J.T., Huang H.L. (2011). Antibacterial properties and human gingival fibroblast cell compatibility of TiO2/Ag compound coatings and ZnO films on titanium-based material. Clin. Oral Investig..

[27] Horandghadim N., Allafi J.K., Urgen M. (2019). Effect of Ta_2_O_5_ content on the osseointegration and cytotoxicity behaviours in hydroxyapatite–Ta_2_O_5_ coatings applied by EPD on superelastic NiTi alloys. Mater. Sci. Eng. C.

[28] Luang H.L., Chang Y.Y., Chen H.J., Chou Y.K., Lai C.H., Chen M.Y.C. (2014). Antibacterial properties and cytocomatibility of tantalum oxide coatings. Surf. Coat. Tech..

[29] Pham V.H., Lee S.H., Li Y., Koh Y.H. (2013). Utility of tantalum (Ta) coating to improve surface hardness in vitro bioactivity and biocompatibility of Co-Cr. Thin Solid Films.

[30] Sui S.Y., Chang J.H., Huang H.H. (2013). Corrosion resistance and biocompatibility of titanium surface coated with amorphous tantalum pentoxide. Thin Solid Films.

[31] Perez I., Sosa V., Perera F.G., Galindo J.T.E., Enriquez-Carrejo J.L., Gonzales P.G.M. (2019). Effect of ion bombardment on the chemical properties of crystalline tantalum pentoxide films. Vacuum.

[32] Koc K., Tepehan F.Z., Tepehan G.G. (2005). Antireflecting coating from Ta_2_O_5_ and SiO_2_ multilayer films. J. Mater. Sci..

[33] Oliver W.C., Pharr G.M. (1992). An improved technique for determining hardness and elastic-modulus using load and displacement sensing indentation experiments. J. Mater. Res..

[34] Lu Y., Zhao W., Yang C., Liu Y., Xiang H., Yang K. (2020). Improving mechanical properties of selective laser melted Co_29_Cr_9_W_3_Cu alloy by eliminating mesh-like random high-angle grain boundary. Mater. Sci. Eng. A.

[35] The Engineering Properties of Co-Cr Alloys (ST2724G) Provided by Phenix Systems. http://brochure.copiercatalog.com/3d-systems/brochure_dentaire_gb.pdf.

[36] Characteristics and Performances of the Phenix Systems PHX Pro200 Dental Equipment Item. http://www.stroumbos.com/index.php/en/products-eng/machines-en/sintering-en/187-phenix-systems-pxs200-en.html.

[37] Zeng Y., Kang L., Wu Y., Wan S., Liao B., Li N., Guo X. (2022). Melamine modified carbon dots as high effective corrosion inhibitor for Q235 carbon steel in neutral 3.5 wt% NaCl solution. J. Mol. Liq..

[38] Wan S., Wei H., Quan R., Luo Z., Wang H., Liao B., Guo X. (2022). Soybean extract firstly used as a green corrosion inhibitor with high efficacy and yield for carbon steel in acidic medium. Ind. Crops Prod..

[39] Ding Z., He Q., Ding Z., Liao C., Chen D., Ou L. (2019). Fabrication and performance of ZnO doped tantalum oxide multilayer composite coatings on Ti6Al4V for orthopedic application. Nanomaterials.

[40] Dkhilalli F., Megdiche S., Guidara K., Rasheed M., Barille R., Megdiche M. (2018). AC conductivity evolution in bulk and greain boundary response of sodium tungstate Na_2_WO_4_. Ionics.

[41] Rasheed M., Barille R. (2017). Room temperature deposition of ZnO and Al: ZnO ultrathin films on glass and PET substrates by DC sputtering technique. Opt. Quantum Electron..

